# Supplementary material for: Engineering ATP import in yeast uncovers a synthetic route to extend cellular lifespan
Source: eLife. 2026 Nov 11;15:RP109761. doi: 10.7554/eLife.109761 (PMC13754279; doi:10.7554/eLife.109761)
Supplement: Peer review history

# Editor report: eLife Assessment

**DOI:** 10.7554/eLife.109761.3.sa0

**Authors:** Felix Campelo

**Affiliations:** Universitat Pompeu Fabra Spain

**Keywords:** Important, Solid

This manuscript addresses an **important** and conceptually ambitious question by using a synthetic biology strategy to perturb ATP homeostasis in yeast and examine its relationship with replicative lifespan. While the lifespan phenotype is robust and the experimental approach is generally **solid**, the current evidence is limited to establish intracellular ATP abundance as the causal determinant of lifespan, particularly given the apparent uncoupling from NTT1-dependent ATP import and single-cell ATP levels, as well as the possibility of extracellular ATP-induced stress responses. Further clarification of the mechanistic interpretation, including more cautious treatment of Snf1/AMPK signaling and NTT1 transport directionality, will be needed before the biological significance of the findings can be fully assessed.

# Referee report: Reviewer #1 (Public review)

**DOI:** 10.7554/eLife.109761.3.sa1

I thank the authors for a thorough revision. The correction to the QUEEN reporter sign convention resolves what was the central issue in my original review: with the reciprocal (1/488 nm) now plotted in Figure 5, the single-cell reporter data and the LC-MS/MS measurements in Figure 2 are internally consistent, and I appreciate the added metabolomics (Figure S2B) and transcriptomics (Figure S2A, S1-S3) that support reduced de novo purine biosynthesis as the source of the lower baseline ATP in NTT1 cells, as well as the quantified Hog1 phosphorylation data (Figure 4C, Figure S8). However, several points from my original review are not yet fully resolved, and I would like the authors to address them before I can recommend acceptance.

(1) A residual, uncorrected statement about QUEEN signal directionality. The Results section (subsection "Altered intracellular ATP level modulates lifespan") still states: "...only 488 nm excitation was used in these experiments, and the resulting QUEEN fluorescence signal is inversely correlated with intracellular ATP levels (Fig. 5A-C)." This directly contradicts the corrected Methods section ("...plotted the inverse of fluorescence measurements (1/488nm), as an indicator of ATP abundance, in Fig. 5, A-C") and the Figure 5 legend and color scale, both of which now correctly state that the plotted quantity increases with ATP abundance. Given that this exact ambiguity was the source of the original contradiction the reviewers raised, I ask the authors to correct this sentence and re-check the rest of the text for any other place where the old (pre-correction) sign convention may still be described.

(2) AMPK/Snf1 validation remains indirect. I had asked for AMPK-pathway controls alongside the Hog1 quantification. The revision addresses this only through transcriptomic inference - downregulation of Snf1 target genes (MIG1, HXK1, HXK2, HXT3; Figure S7) is used to argue for low Snf1 activity under high ATP. This is a reasonable correlative observation but is not a direct validation of Snf1/AMPK activity (e.g., Snf1 Thr210 phosphorylation status or a kinase activity assay), which is what I intended by "AMPK controls." Please either add a direct biochemical readout of Snf1 activation status analogous to the Hog1 blots or explicitly temper the claims about Snf1-mediated metabolic rewiring to reflect that they rest on transcriptomic inference alone.

(3) Bidirectional-transport hypothesis addressed indirectly, not experimentally. The new metabolomics (ATP/ADP/AMP all reduced in untreated NTT1 cells) and the coordinated downregulation of the purine biosynthesis pathway are a coherent case for "reduced synthesis" as the parsimonious explanation, and I appreciate this analysis. However, this remains a correlative/transcriptomic argument rather than the direct flux experiment I suggested was feasible (e.g., an isotope-labeled ATP efflux/uptake assay to directly test whether NTT1 exports as well as imports ATP). A contraction of the adenylate pool via reduced synthesis and a bidirectional transporter are not mutually exclusive, and the current data cannot fully distinguish them. I would ask the authors to either perform such a direct test or explicitly acknowledge this as a limitation and an alternative that current data do not rule out.

In summary, the revision has substantially improved the manuscript and resolved the central ATP-directionality contradiction, but points 1-3 above should be addressed, particularly point 1, since it re-introduces the same ambiguity, the revision was meant to eliminate, before I can consider my concerns fully resolved.

# Referee report: Reviewer #2 (Public review)

**DOI:** 10.7554/eLife.109761.3.sa2

Summary:

This work presents interesting findings where addition of exogenous ATP extends replicative lifespan of yeast cells in a way that seems uncorrelated with actual increased intracellular ATP levels or mitochondria. To be clear, addition of ATP to yeast growth media increases the number of cell divisions per cell in yeast. Expression of the NTT1 ATP transporter gene increases intracellular ATP levels according to LCMS analysis, but the effect on replicative lifespan works without the NTT1 gene and without an intracellular increase in ATP (possibly with a decrease in intracellular ATP), so the effect appears to be independent of the effect on intracellular ATP levels or mitochondria, as mitochondria-less R0 yeast cells also have increased numbers of cell division when grown with extracellular ATP. I would not state that the data support a role for ATP availability regulating aging because human cells do not import ATP from the environment; maybe for yeast or microbes that import ATP unlike normal human cells that make all their own. I would not state that the data support a role for ATP in increasing replicative lifespan since there is no correlation between increased lifespan an increased ATP- rather the opposite is shown - you got no correlation between intracellular ATP at replicative lifespan. What you show is that you can increase replicative lifespan when you increase extracellular ATP in a way that doesn't matter if you further increase intracellular ATP with NTT1, but maybe you were saturated. But then, intracellular ATP doesn't correlate either. So, it doesn't make sense to say that the paper shows that increasing ATP increases replicative lifespan. You gave the cells what seems like an osmotic stress and it didn't even matter if they had the mitochondria to respond. Lifespan didn't correlate with more ATP at all. So, it seems incorrect to indicate that ATP regulates lifespan in yeast, when you haven't shown that it just wasn't osmotic stress and you have shown that it doesn't really have anything to do with how much ATP each cell has. Rather, it seems more like it's the robustness of some other response to the ATP and not how much they imported.

The authors point out in their rebuttal that, paraphrased, with the QUEEN reporter gene that quantifies ATP via ratiometric fluorescence, some cells increase atp with age, some cells decrease it and others show no age related changes, so it would not be meaningful to make an ATP measurement in old cells. Essentially, that line of reasoning further supports that, while adding extracellular atp seems to increase replicative lifespan, the lack of correlation between intracellular ATP and replicative aging suggest that they are eliciting a stress response to having high extracellular ATP. The increased replicative lifespan seems to be caused by some kind of stress response to the extracellular ATP, possibly osmotic. So, this is an interesting result that suggests that there are mechanisms to respond to high extracellular ATP that result in increased replicative lifespan that aren't caused by the ATP per say, but by having a high concentration of ATP, outside the cell. This paper seems like it might relate to the Kaeberlein paper about high osmolarity increasing replicative lifespan, but I don't know if this condition qualifies as high osmolarity.

As an aside, this seems like a better model for increased tumor cell growth in the presence of increased extracellular ATP, which happens in some cancers.

Strengths:

Longitudinal imaging of single cells. Analyzed ATP levels with two approaches. Creative approach to use NTT1 transporter to increase intracellular ATP levels (that did nothing for lifespan). Solid replicative lifespan data. Seemingly clear lack of correlation between ATP and replicative lifespan among individual cells strongly pointing so some kind of stress response, possibly some kind of osmolarity response like Kaeberlein 2002.

Weaknesses/inconclusive data:

The LCMS data seems better than the microscopy data on ATP because it does not suffer from signal interference/befuddlement from autofluorescence or pH or age-related changes in either of those parameters. The authors seem to have subtracted an average autofluorescence from each cell in their revised analysis, but it is unclear if this value changes with age to account for age related changes in autofluorescence; it is also unclear if signal attenuation as a function of age-related intracellular pH change was accounted for. They may have age-related values for autofluorescence or age-related changes in intracellular pH that affect fluorescence of GFP if they go back to their raw data, which might be useful for signal cleanup.

# Author comment: Author response

**DOI:** 10.7554/eLife.109761.3.sa3

**Authors:** Naci Oz, Hetian Su, Vedat Sari, Paraveen Patnaik, Rohil Hameed, Jong Hee Song, Derek C Prosser, Vyacheslav M Labunskyy, Vadim N Gladyshev, Nan Hao, Alaattin Kaya

**Affiliations:** Virginia Commonwealth University Richmond United States; University of California, San Diego La Jolla United States; University of Virginia Charlottesville United States; Boston University School of Medicine Boston United States; University of Virginia Charlottesville United States; University of Virginia Charlottesville United States; Virginia Commonwealth University Richmond United States; Boston University School of Medicine Boston United States; BRIGHAM WOMENS HOSPITAL Boston United States; University of California, San Diego La Jolla United States; University of Virginia Charlottesville United States

The following is the authors’ response to the original reviews.

Summary of the Key Clarification Underlying This Revision

Both reviewers independently identified what appeared to be a direct contradiction between our LC-MS/MS measurements of intracellular ATP (Figure 2) and our single-cell QUEEN reporter measurements of intracellular ATP (Figure 5): NTT1 expression appeared to lower rather than raise intracellular ATP, and exogenous ATP treatment appeared to further lower the QUEEN signal even though LC-MS/MS showed the opposite. We have carefully reviewed the reviewers’ comments alongside our original figures and legends, and we agree that the source of this apparent contradiction was insufficiently explained in the original submission.

To make the reported quantity intuitively track ATP abundance, we have now:

- Plotted the reciprocal of the 488 nm signal (1/488 nm) in the updated Figure 5, eliminating the inverse correlation between AU values and ATP abundance;

- Revised the Figure 5 legend, axis labels, Methods, and Results to explicitly state that the plotted value is directly proportional to intracellular ATP abundance, removing any ambiguity about directionality;

- Subtracted autofluorescence from the raw QUEEN reporter fluorescence signal;

- Expanded the Methods and Results to explain, side by side, why the QUEEN-based and LC-MS/MS based measurements are complementary but not numerically identical (different dynamic ranges; live single-cell sampling over time versus bulk lysates at fixed timepoints); and

- Re-examined Figures 2, 5, and 7 together to confirm that, once the QUEEN directionality is correctly read, the two datasets are internally consistent: NTT1 expression lowers baseline intracellular ATP relative to Wt, and exogenous ATP supplementation raises intracellular ATP in NTT1 cells (and, more modestly and via a distinct mechanism, in Wt cells), by both methods.

Because this misunderstanding affected interpretation of the foundational ATP data, it appears to have influenced evaluation of all downstream conclusions. For example, neither reviewer meaningfully engaged with:

- The identification of distinct cell death trajectories of aged cells;

- The mitochondrial dependency of NTT1-associated toxicity and the observed lifespan differences; and

- The distinction between intracellular ATP manipulation and extracellular ATP sensing mechanisms.

Example of a Data Discrepancy Resolved by This Clarification

In some instances, we think the original figures did not present the data clearly enough to support the intended reading, which we address below.

**Public Reviews:**

**Reviewer #1 (Public review):**

Summary:

The authors aim to engineer a synthetic system for manipulating ATP homeostasis in budding yeast by expressing the microsporidian nucleotide transporter NTT1, thereby enabling ATP import from the extracellular environment. Using this system, they attempt to test whether intracellular ATP abundance causally regulates replicative lifespan and whether extracellular ATP sensing contributes independently to longevity pathways. The manuscript presents data from ATP biosensing, transcriptomics, mitochondrial perturbations, and microfluidic aging assays to build a dual-mechanism model linking ATP availability, MAPK signaling, mitochondrial function, and aging trajectories.

Strengths:

A major strength of the study is its creative application of xenotopic synthetic biology to directly manipulate ATP homeostasis-an ambitious approach that addresses an important and difficult question in aging biology. The use of complementary methods, including single-cell ATP reporters, microfluidic lifespan measurements, and RNA-seq, generates a rich experimental dataset with the potential to reveal multiple layers of ATP-dependent physiological regulation. The manuscript also raises interesting hypotheses regarding extracellular nucleotide sensing and HOG/MAPK pathway involvement, opening conceptual space for future exploration of ATP-based signaling in yeast.

Weaknesses:

Despite these strengths, the manuscript suffers from several critical weaknesses that undermine the central conclusions. Foremost, the intracellular ATP measurements contradict key interpretations: NTT1 expression lowers ATP levels, yet multiple sections assert or assume that NTT1 increases intracellular ATP via import. This unresolved contradiction propagates throughout the mechanistic model. The authors do not consider or experimentally address the more parsimonious explanation that NTT1 may be a bidirectional ATP transporter, which would unify many perplexing results. Several important analyses are missing (e.g., transcriptomic comparison of NTT1 cells with vs. without ATP), and key signaling claims lack proper validation (e.g., Hog1 quantification, AMPK controls). Additionally, inconsistencies in figures-such as incorrect scale bars, mismatched ATP measurements, and a conceptual model contradicted by the data-further detract from clarity. As a result, the manuscript does not yet convincingly achieve its stated aims, and the current evidence does not adequately support the proposed causal relationships between ATP homeostasis and lifespan.

We thank the reviewer for a rigorous and constructive critique. The central objection that our intracellular ATP measurements contradict our interpretation arose from a presentational error in how the QUEEN reporter data were plotted. As described in our Methods (QUEEN reporter section), the QUEEN biosensor is a ratiometric reporter whose 410 nm/488 nm excitation ratio increases with ATP binding. Because repeated 410 nm excitation causes phototoxicity over the multi-day time course of a replicative aging experiment, our longitudinal single-cell measurements (Figure 5) used only the 488 nm channel, for which fluorescence intensity decreases as ATP binds the reporter. In the original Figure 5, arbitrary fluorescence units (AU) were therefore inversely correlated with intracellular ATP abundance, such that higher AU values corresponded to lower ATP levels. We described this inverse relationship in the Results section and labeled the relevant figure panels “Low versus High,” but on reflection we agree this was not stated clearly enough in the figure legend itself, where a reader would most naturally look for it. As a result, the two datasets read as contradictory even though they are fully consistent, and we take responsibility for that lack of clarity.

The reviewer proposes that NTT1 may function as a bidirectional transporter, with net ATP export accounting for the reduced ATP we observe in untreated *NTT1*-expressing cells. We considered this possibility, and our expanded metabolite data argue against it. LC-MS/MS shows that ATP, ADP and AMP are all significantly reduced in untreated *NTT1*-expressing cells relative to wild type (new Figure S2B), with AMP most severely affected a contraction of the entire adenylate pool rather than a shift in its distribution.

Our transcriptome data identify the source of this contraction. *NTT1* expression produces a coordinated downregulation of de novo purine biosynthesis, spanning the pathway (new Figure S2A), consistent with the pronounced AMP deficit. Parallel pyrimidine biosynthesis genes are unaffected, indicating a purine-specific response rather than a general biosynthetic shutdown. We therefore conclude that reduced de novo purine synthesis, not reverse transport, is the parsimonious explanation for the lower baseline nucleotide levels in *NTT1*-expressing cells, and we have added this analysis and interpretation to the Results and Discussion.

Under ATP-replete conditions, both LC-MS/MS and the corrected QUEEN readout show a clear increase in intracellular ATP in *NTT1*-expressing cells, consistent with import. We have additionally added the transcriptomic comparison of *NTT1*-expressing cells with and without exogenous ATP that the reviewer requested (new Figures S1–S3), quantified Hog1 phosphorylation, and audited every figure, legend and numerical value in the manuscript.

**Reviewer #2 (Public review):**

Summary:

This work presents interesting findings where the addition of exogenous ATP extends the replicative lifespan of yeast cells in a way that seems uncorrelated with actual increased intracellular ATP levels or mitochondria. To be clear, the addition of ATP to yeast growth media increases the number of cell divisions per cell in yeast. Expression of the NTT1 ATP transporter gene increases intracellular ATP levels according to LCMS analysis, but the effect on replicative lifespan works without the NTT1 gene and without an intracellular increase in ATP (possibly with a decrease in intracellular ATP), so the effect appears to be independent of the effect on intracellular ATP levels or mitochondria, as mitochondria-less R0 yeast cells also have increased numbers of cell division when grown with extracellular ATP. The plots in Figure 5 make it seem like exogenous ATP addition lowers intracellular ATP for both the NTT1 cells and the wild-type cells, and that is not what the data in Figure 2d with LCMS shows.

As an aside, this seems like a better model for increased tumor cell growth in the presence of increased extracellular ATP, which happens in some cancers.

Restated, the data suggest they were successful in increasing intracellular ATP by LCMS, but not by queen reporter, and that the seemingly likely increased intracellular ATP was not causative, as cells that did not have an increase in intracellular ATP, but had the same exogenous ATP addition, also gained an increase in replicative lifespan. There could also be two distinct mechanisms extending replicative lifespan to the same degree in these two different strains. More measurements, controls, and analyses are needed to accurately determine what is happening with intracellular ATP levels with age. It is currently unknown if there is any correlation between ATP levels and replicative aging (with properly controlled longitudinal measurements).

Strengths:

Longitudinal imaging of single cells. Analyzed ATP levels with two approaches. Creative approach to use NTT1 transporter to increase intracellular ATP levels. Solid replicative lifespan data.

Weaknesses:

Mostly unclear about ATP levels with age and the relationship, or lack thereo,f between intracellular ATP levels and replicative lifespan. No idea what this effect depends on, but some ideas what it does not depend on (mitochondria or increased intracellular ATP). Experiments seem to lack biological controls (cells without gfp) for age related changes in autofluorescence (and pH that can affect gfp signal) for the fluorescent microscopy quantifying ATP with age using the QUEEN reporter (seems that way as written); conflicting evidence on ATP levels; lack of LC-MS measurements in old cells; no apparent correlation between ATP levels and replicative lifespan, but that could be wrong - just not apparent from the longitudinal data plots. The LCMS data seems better than the microscopy data on ATP because the microscopy approach seems to lack proper biological controls, and the selection of only the top 40% of pixels to quantify signal seems unjustified as written, and possibly prone to technical artifacts. Figure 2 B&C plots of ATP levels should show what the cells were normalized to. The figures also seem too diluted and should probably be combined or put in the supplements (hog1 western) if they do not relate to the lifespan effect. There seem to be some technical scientific editorial errors, like in Figure 7.

We appreciate this careful and, in most respects, accurate reading. The reviewer concludes that the lifespan extension conferred by extracellular ATP does not depend on an increase in intracellular ATP, since it occurs in cells lacking NTT1 and in rho^0^ cells, and suggests that two distinct mechanisms may extend lifespan in the two strains. This is precisely the model our data supports, and we are encouraged that the reviewer arrived at it independently: we propose an intracellular, import-dependent and mitochondria-dependent mechanism operating in *NTT1*-expressing cells, and a separate extracellular ATP-sensing mechanism that acts without import and without functional mitochondria. We have rewritten the Results and Discussion to make this dual-mechanism structure explicit rather than leaving it to be inferred, and Figure 8 has been revised so that the model matches the data.

The apparent conflict between the QUEEN and LC-MS/MS measurements, which underlies much of the reviewer's uncertainty about ATP levels with age, is resolved by the correction described below. We have additionally subtracted autofluorescence from the reporter signal, stated the normalization for the ATP measurements in the Figure 2 legends, and added a justification in the Methods for restricting quantification to the brightest pixels. The QUEEN reporter is a soluble cytoplasmic protein excluded from the vacuole, which occupies an increasing fraction of cell volume in ageing mother cells, so whole-cell quantification systematically underestimates cytoplasmic signal in old cells for reasons unrelated to ATP. On the request for LC-MS/MS on sorted old cells, we agree this would be valuable in principle, but because individual cells follow divergent ATP trajectories with age, a bulk measurement of a sorted population would average across those trajectories and would not be directly comparable to our single-cell data.

**Recommendations for the authors:**

**Reviewer #1 (Recommendations for the authors):**

The intracellular ATP measurements contradict key interpretations: NTT1 expression lowers ATP levels, yet multiple sections assert or assume that NTT1 increases intracellular ATP via import. This unresolved contradiction propagates throughout the mechanistic model. The authors do not consider or experimentally address the more parsimonious explanation that NTT1 may be a bidirectional ATP transporter… Several important analyses are missing (e.g., transcriptomic comparison of NTT1 cells with vs. without ATP), and key signaling claims lack proper validation (e.g., Hog1 quantification, AMPK controls). Additionally, inconsistencies in figures, such as incorrect scale bars, mismatched ATP measurements, and a conceptual model contradicted by the data, further detract from clarity.

We thank the reviewer for this central and constructive critique. As explained above, the apparent contradiction in intracellular ATP directionality stemmed from an underexplained sign convention in the QUEEN reporter readout in Figure 5, which we have now corrected and clarified throughout the text, legends, and axis labels. With this correction, NTT1 cells show reduced baseline ATP and a clear, dose-dependent increase in intracellular ATP upon exogenous ATP treatment, consistent between the QUEEN and LC-MS/MS assays. There is a discrepancy between the two methods (QUEEN versus LC-MS/MS) specifically in the Wt cell ATP measurements, which we discuss in the Results section.

The authors seem to assume that NTT1 expression should result in ATP influx… the authors should consider an alternative interpretation: the ATP transporter is bidirectional… this interpretation seems to be consistent with many of the observations in the manuscript, and this bidirectional ATP flux can be experimentally tested.

We thank the reviewer for this insightful alternative hypothesis. With the QUEEN directionality now corrected (see summary above), the core observation motivating this suggestion lower ATP in NTT1 cells alongside evidence of import is substantially resolved: NTT1 cells have lower baseline intracellular ATP than Wt, and both LC-MS/MS and the corrected QUEEN readout show that exogenous ATP raises intracellular ATP specifically in NTT1 cells, consistent with net import under ATP-replete conditions. In addition, our expanded LCMS/MS data (Figure S2) suggest that NTT1 overexpression results in decreased de novo purine biosynthesis, offering a possible explanation for the decreased ATP levels in these cells.

The RNA-seq analysis compared the effects of adding exogenous ATP in WT and NTT1 cells. However, analysis can also be done to compare the effects of NTT1 under conditions with or without exogenous ATP.

We have added the missing transcriptomic comparison of NTT1 cells with versus without exogenous ATP, and the corresponding data are now presented in three new supplementary figures (Figures S1–S3).

“In NTT1-expressing cells, ATP is directly imported from the extracellular environment…” the authors are assuming NTT1 cell imports ATP from media with ATP supplementation. This should be reconciled with the lower intracellular ATP concentration and lifespan shortening.

This statement refers specifically to the ATP-treated condition, in which both LC-MS/MS and the corrected QUEEN measurements show elevated intracellular ATP in NTT1 cells relative to untreated NTT1 cells and to Wt. We have revised the text to make this conditional context explicit.

The western blots for Hog1p and Hog1 should be quantified… I suggest using p-Hog1 or phos-Hog1 to indicate the phosphorylated form.

We have now quantified Hog1 phosphorylation by western blot analysis from two independent biological replicate experiments, each probed with a different commercial phospho-Hog1 antibody, to confirm that the result is not antibody-specific (Figure 4, Figure S8; Methods). We have also adopted the reviewer’s suggested nomenclature and now consistently denote the phosphorylated form as Hog1ᵖ throughout the text and figures to avoid ambiguity with the Hog1p gene product naming convention.

The model in Figure 8 indicates a larger lifespan extension in the NTT1 cells. This is not consistent with the data presented in Figure 5-7.

The statement regarding a larger lifespan extension is consistent with the data presented in Figures 5 and 7, but we agree this connection was not spelled out clearly enough in the original text. These two figures differ in their treatment conditions. In Figure 6D, 10 µM ATP treatment resulted in a significant median lifespan extension in both Wt and NTT1 cells, though the magnitude of the extension differed: As stated in the Results, Wt cells showed a median RLS increase of 41% (p = 1.28E-12) upon 10 µM ATP supplementation, while NTT1 cells, which also showed an increase in ATP levels (Fig. 6B, C), showed an even more pronounced extension (median RLS increase = 60%, p = 4.03E-18) in lifespan (Fig. 6D). In Figure 7, we analyzed the higher dose of ATP treatment (10 mM) to examine its toxic effect on lifespan. Figure 7A shows that the lifespan-extension effect observed at the low ATP dose (Fig. 6) diminished under the 10 mM treatment condition for NTT1 cells but not for Wt cells. Figure 7B shows that elimination of functional mitochondria rescues this mitochondrial toxicity in NTT1 cells, such that these cells extend their lifespan to a magnitude comparable to Wt under 10 mM ATP treatment conditions.

**Reviewer #2 (Recommendations for the authors):**

The fluorescence imaging shows that cells with exogenous ATP have lower ATP levels, and that is not what was shown in Figure 2 by LCMS. So, as it is, this is confusing and not conclusive and may have to do with technical artifacts.

We would like to clarify that our data do not in fact conflict. Figure 2B (fluorescence imaging) shows that NTT1 cells have lower intracellular ATP than Wt under baseline (no exogenous ATP) conditions, and that exogenous ATP treatment increases intracellular ATP abundance in both cell types. Figure 2C shows that under 2-deoxyglucose (2DG) treatment, Wt cells display decreased ATP levels regardless of exogenous ATP treatment, whereas NTT1 cells show increased intracellular ATP upon ATP treatment, further supporting the import function of NTT1. Our LC-MS/MS data (Fig. 2D, left panel) independently corroborate the fluorescence results in Figure 2B: NTT1 cells show lower ATP abundance than Wt cells at baseline, and exogenous ATP treatment raises intracellular ATP in NTT1 cells but not in Wt cells. We discuss the modest discrepancy between the LC-MS/MS and QUEEN measurements specifically for Wt cells in the Results section.

The plots in Figure 5 make it seem like exogenous ATP addition lowers intracellular ATP for both the NTT1 cells and the wild-type cells, and that is not what the data in Figure 2d with LCMS shows… Restated, the data suggest they were successful in increasing intracellular ATP by LCMS, but not by queen reporter, and that the seemingly likely increased intracellular ATP was not causative…

We appreciate the reviewer’s detailed reading of Figures 2 and 5. As described above, this apparent conflict arose because Figure 5 originally plotted a raw fluorescence value that is inversely related to ATP abundance without stating this clearly enough in the legend. We have corrected the legend, axis labels, and Results text so that the plotted quantity in Figure 5 (1/488 nm QUEEN signal) increases with ATP abundance, matching the LC-MS/MS data in Figure 2. With this correction, both assays agree that exogenous ATP treatment raises intracellular ATP in NTT1 cells; we discuss in the Results the more modest and assay-dependent behavior we observe in Wt cells, where LC-MS/MS and QUEEN differ somewhat in sensitivity and time-resolution.

It needs a bit more experimental clarification and some thinking about what it means to disrupt ATP homeostasis with high extracellular levels of ATP, a generally biologically unnatural/pathological situation.

The central premise of our study is that altered intracellular ATP is associated with lifespan. Mitochondrial functional decline is a hallmark of ageing, but whether this correlates with decreased ATP, and whether age-associated ATP changes are linked to cellular aging, remains an overlooked question. Here, for the first time, we show that not every cell exhibits a decreasing ATP trajectory with age, and that maintaining ATP homeostasis can extend lifespan. Interestingly, extracellular ATP sensing is directly relevant to cell-to-cell communication (purinergic signaling); however, this has never been characterized in yeast. Our data indicate that a similar, conserved mechanism may also be present in yeast, although the exact components of this sensing cascade require further study. This information is included in the Introduction and Discussion sections of the manuscript.

The data for ATP levels with age needs to be cleaned up/refined… Sort some high bud scar cells and test them with LCMS… run wild-type cells to subtract out the autofluorescence… or redo it on a scope with linear unmixing.

Our data indicates that each cell follows a different ATP-abundance trajectory during aging: while some cells show decreasing ATP with age, others show no change, and still others show increasing ATP. Population-level testing of sorted old cells would therefore not yield results that are directly comparable to our microfluidic experiments, in which we track individual cell trajectories over time. In the updated figure, we have subtracted autofluorescence.

Figures 1 and 2 could be combined, and so could 3 and 5, 6 and 7. Figure 4 should probably be a supplement or part of another figure since upregulating HOG1 has nothing to do with the lifespan extension effect.

We appreciate this suggestion for streamlining the figures. Our data show only decreased Hog1 activity under ATP treatment conditions; we did not test whether this decline in Hog1 activity is associated with lifespan extension. However, we would like to include this figure in the main text, because it directly validates the effect of extracellular ATP sensing on modulation of Hog1 activity.

Figure 7 seems like it is showing that the NTT1 strain’s shorter replicative lifespan is rescued by losing mitochondria, and not that toxicity from ATP in the NTT1 is alleviated by losing mitochondria.

Both the NTT1 strain’s shorter replicative lifespan and its ATP toxicity (10 mM) are rescued by eliminating respiratory mitochondria. In Figure 7, we analyzed the higher dose of ATP treatment (10 mM) to examine its toxic effect on lifespan. Figure 7A shows that the lifespan-extension effect observed at the low ATP dose (Fig. 5) diminished under the 10 mM treatment condition for NTT1 cells but not for Wt cells. Figure 7B shows that elimination of functional mitochondria rescues this mitochondrial toxicity in NTT1 cells, such that these cells extend their lifespan to a magnitude comparable to Wt under 10 mM ATP treatment conditions.

I think you should make sure your pinhole is opened up to catch all the signal in z from each cell… It seems like taking the mean intensity of the top 40% of pixels in a cell can create technical artifacts… Please justify why you use that approach.

We have added justification for the top-40%-pixel approach to the Methods: because the QUEEN reporter is a soluble cytoplasmic protein excluded from the large vacuoles that occupy an increasing fraction of cell volume as mother cells age, whole-cell or full-z-stack quantification systematically underestimates cytoplasmic ATP signal in older cells for reasons unrelated to ATP abundance. Restricting analysis to the brightest pixels focuses quantification on reporter-containing cytoplasm and avoids this age-confounded dilution artifact.
